# Positive fluid balance as an early biomarker for acute kidney injury: a prospective study in critically ill adult patients

**DOI:** 10.6061/clinics/2021/e1924

**Published:** 2021-02-01

**Authors:** Maria Olinda Nogueira Ávila, Paulo Novis Rocha, Caio A. Perez, Tássia Nery Faustino, Paulo Benigno Pena Batista, Luis Yu, Dirce Maria T. Zanetta, Emmanuel A. Burdmann

**Affiliations:** I LIM 12, Disciplina de Nefrologia, Faculdade de Medicina FMUSP, Universidade de Sao Paulo, Sao Paulo, SP, BR; II Monte Tabor Hospital Sao Rafael, Salvador, BA, BR; III Medicina Interna e Apoio Diagnostico, Universidade Federal da Bahia, Salvador, BA, BR; IV Departamento de Epidemiologia, Faculdade de Saude Publica, Universidade de Sao Paulo, Sao Paulo, SP, BR; V Universidade do Estado da Bahia, Salvador, BA, BR; VI Escola Bahiana de Medicina e Saude Publica, Salvador, BA, BR; VII Faculdade de Medicina, Uniao Metropolitana de Educacao e Cultura UNIME/KROTON, Lauro de Freitas, BA, BR

**Keywords:** Acute Kidney Injury, Biomarker, Positive Fluid Balance, Water Balance, Intensive Care Unit, Mortality

## Abstract

**OBJECTIVES:**

Positive fluid balance is frequent in critically ill patients and has been considered a potential biomarker for acute kidney injury (AKI). This study aimed to evaluate positive fluid balance as a biomarker for the early detection of AKI in critically ill patients.

**METHODS:**

This was a prospective cohort study. The sample was composed of patients ≥18 years old who stayed ≥3 days in an intensive care unit. Fluid balance, urinary output and serum creatinine were assessed daily. AKI was diagnosed by the Kidney Disease Improving Global Outcome criteria.

**RESULTS:**

The final cohort was composed of 233 patients. AKI occurred in 92 patients (40%) after a median of 3 (2-6) days following ICU admission. When fluid balance was assessed as a continuous variable, a 100-ml increase in fluid balance was independently associated with a 4% increase in the odds of AKI (OR 1.04; 95% CI 1.01-1.08). Positive fluid balance categorized using different thresholds was always significantly associated with subsequent detection of AKI. The mixed effects model showed that increased fluid balance preceded AKI by 4 to 6 days.

**CONCLUSION:**

These results suggest that a positive fluid balance might be an early biomarker for AKI development in critically ill patients.

## INTRODUCTION

Acute kidney injury (AKI) is a worldwide public health problem that adversely affects patients’ short- and long-term outcomes (1-6). The identification of high-risk individuals and early diagnosis are crucial to avoid it or minimize its severity (7-10). The current AKI definitions use high serum creatinine (SCr) and/or low in urinary output (UO) as diagnostic criteria (9-10). A UO <0.5 ml/kg/h for six hours diagnoses AKI according to those definitions. However, a UO over this cutoff might be insufficient to keep up a neutral fluid balance (FB) in critically ill patients, who usually receive large amounts of fluid and can develop a positive FB despite having a “normal” UO. In this circumstance, if SCr has not risen, the patient would not meet the AKI criteria, despite having a clear impairment in the kidneys’ ability to remove the fluid load.

Healthy kidneys have remarkable dilution ability and can eliminate up to 18 liters of maximally diluted urine/day (11). This ability depends on the functional integrity of the medullary microcirculation, which is impaired during the early stages of kidney disease (12). Thus, a positive FB might be an early biomarker for AKI, preceding changes in UO and SCr (13-16). This prospective cohort study aimed to evaluate the possible association between early positive FB and subsequent AKI development in critically ill patients.

## METHODS

This study was prospective and observational, with a single-center cohort. This protocol was approved by the Institutional Review Board of the Monte Tabor-Hospital São Rafael, Brazil (protocol 37/11). An informed consent waiver was granted due to the observational nature of the study.

### Study population and site

Inclusion criteria*:* ≥18 years old, admitted to a clinical/surgical ICU from August 2011 to July 2013.

Exclusion criteria: CKD stage 5, AKI at ICU admission, obstructive AKI, transient AKI, solid organ and/or bone marrow transplant, pregnancy, terminal cancer, ICU stay less than three days and incomplete medical records or files.

### Groups studied

AKI group: patients who developed AKI during ICU hospitalization.

Non-AKI group: a control group composed of patients without AKI. For each AKI case, we randomly drafted a control from a pool of non-AKI patients who had a length of ICU stay ≥ than the time to AKI of the corresponding AKI patient. For example, for a patient developing AKI on ICU day seven, we randomly drafted a control from the pool of non-AKI patients who had a length of ICU stay ≥ seven days. For such a case, we compared the FB of the first six days of the ICU stay of the control with the FB of the six days preceding AKI in the patient.

### Analyzed parameters

At admission: age, sex, comorbidities (diabetes mellitus, arterial hypertension, cancer, and heart disease reported in the medical file), and the Acute Physiology and Chronic Health Evaluation (APACHE) II score.

Daily: hourly UO, FB, vasoactive drug use (noradrenaline, dopamine, or vasopressin), mechanical ventilation use, and serum test results for creatinine, transaminases, total and direct bilirubin, prothrombin time (PT) and activated partial thromboplastin time (APTT).

The primary endpoint was AKI development.

### Fluid balance

The daily FB was calculated using the formula: FB = Total Daily Intake (all fluids received by the patient) - Total Daily Output (all fluids eliminated by the patient). It was evaluated as a continuous or as a categorical variable. When assessed as a categorical variable, we used the quartiles, using cut points estimated from all daily measurements of the days preceding AKI and the corresponding days of the control group together. We also categorized FB as < zero, zero to 1,500 and >1,500 ml/24h using the mean FB of daily measurements of the days preceding AKI and the corresponding days in the control group for each individual patient.

### Definitions

AKI was defined by the KDIGO criteria considering both SCr and UO (10).

Transient AKI was defined as AKI that decreased the SCr and/or increased the UO within 24 to 48h after volume replacement and/or the use of vasoactive drugs (10).

Sepsis was defined by the International Sepsis Definitions Conference (17).

Shock was defined as the use of vasoactive drugs to maintain a mean arterial blood pressure >60 mmHg (17).

Respiratory failure was defined as the use of invasive mechanical ventilation (17).

Liver dysfunction was defined as PT <50% and a serum bilirubin and transaminase level increase to ≥3 times the ICU admission values (17).

A coagulation disorder was defined as an APTT ≥1.5 times the value at ICU admission (without using heparin), PT <50% and a platelet count <150,000 (17).

### Statistical analysis

We used the Statistical Package for the Social Sciences for Windows (SPSS) version 17.0 (SPSS Inc., Chicago, Il, USA) and R version 3.1.1 (R Foundation for Statistical Computing, Vienna, Austria). Variables are presented as the mean and standard deviation, median and interquartile range (IQR), or simple and relative frequencies. Comparisons of continuous variables between two groups were conducted using Students t test or the Mann-Whitney test; comparisons of categorical variables were performed using the chi-square or Fisher’s exact test.

We used multiple logistic regression analysis to evaluate the effect of FB as an independent variable on the incidence of AKI. In these analyses, we used the FB of the days prior to AKI detection in the patient and the corresponding days in the drafted controls. Goodness of fit was evaluated with the Hosmer-Lemeshow test. The same models were fitted with FB as a continuous or categorized variable. Its evaluation as a continuous variable allows the estimation of the average strength of association over its entire range, and as a categorized variable, it gives a measure of an effect that is easier to interpret. The models included variables with *p*<0.20 in the univariate analysis and variables considered clinically relevant: age; APACHE II score; presence of arterial hypertension, cancer, heart disease, sepsis, mechanical ventilation, coagulopathy, and liver dysfunction; and use of vasoactive drugs. The following models were evaluated:

Model 1: This model compared the mean FB until AKI development in the patients against the mean FB of the corresponding days in the drafted controls.

Model 2: All daily FBs were assessed until AKI development, and those of the corresponding days in the drafted controls were used to estimate the cut points to categorize mean FB before AKI in quartiles.

Model 3: FB was assessed in the two days prior to AKI detection in the cases, and the corresponding days in the drafted controls were grouped to estimate the cut points to categorize the mean FB of the two days before AKI into quartiles. In both models, the first quartile was used as the reference group.

Model 4: The thresholds of the mean FB in the days preceding AKI detection and the mean of the corresponding days in the controls were compared as follows: mean FB <zero/24h, FB >1,500 ml/24h and 0≤FB≤1,500 ml/24h (reference group).

The magnitude of the associations was estimated using odds ratios and their respective 95% confidence intervals (95% CI).

To estimate the change in FB with time before AKI, the mean FB of days 9 to 7, 6 to 4 and 3 to 1 before AKI and of the respective days for the control group were evaluated by a mixed effect model, considering the repeated measurements in each patient. Fixed effects were the three periods cited above, with the period of -9 to -7 days as a reference, and the presence of AKI. The interaction of these two variables was tested to see if FB changed differently over time in patients with *vs*. without AKI. The model was adjusted for sex, age, APACHE II score, presence of sepsis, heart disease, liver dysfunction, mechanical ventilation and vasoactive drugs. Subject effects were assumed to be random. We considered the random errors and effects to be independent and normally distributed. Upon residual analysis, this assumption of the normally distributed response was valid. The mixed model assumptions were tested using a graphical method of residual distribution with fitted values and random effects estimated values. The significance of the fixed effects of the model was evaluated using the Wald test. A *p*-value <0.05 was considered statistically significant.

As a sensitivity analysis, we repeated the mixed effect model using a mean FB of 4 to 6 days (reference) and 3 to 1 days before AKI because the mean FB of days 9 to 7 before AKI was estimated in fewer than half of the patients, since the median time to AKI was three days. The model was adjusted for the same variables included in the previous one.

Also, as a sensitivity analysis, these statistical analyses were performed while including transient AKI patients, and corresponding non-AKI patients were included in the control group. As an additional sensitivity analysis, a polynomial regression was used to evaluate the association of FB, as an independent variable, and AKI. The reference category for this analysis was the non-AKI group, contrasting with the transient AKI and AKI groups (those included in the study). The model was adjusted for the same variables included in the logistic regression analysis.

## RESULTS

**Figure 1:**
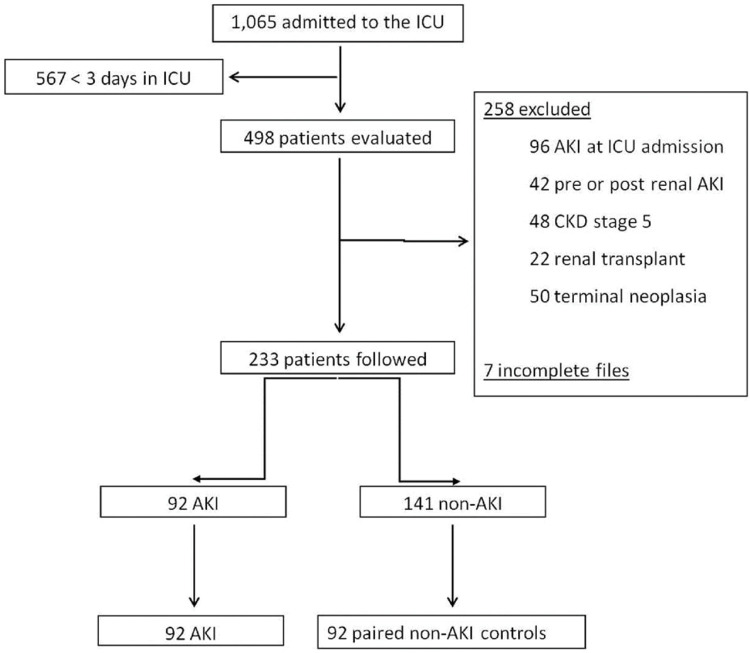
Study flowchart.

A total of 1,065 adult patients were hospitalized in the ICU during the study; 567 had an ICU stay <three days and were not included. Of the 498 remaining patients, 258 were excluded (96 with AKI at ICU admission, 29 with transient AKI, 13 with obstructive AKI, 48 with stage 5 CKD, 22 renal transplant recipients, and 50 terminally ill cancer patients), and seven were not evaluated due to incomplete files. The final cohort was composed of 233 patients (Figure 1).

AKI occurred in 92 patients (40%) after a median of 3 (2-6) days following ICU admission, and 60% were in KDIGO stage I.

Considering the entire ICU stay, the mean FB for the 233 patients was 834±847 ml/day (median 739 ml/day, IQR 253 - 1,343 ml/day; minimum -2,302 ml/day and maximum 3,457 ml/day).

In Table 1, we present the entire population’s clinical characteristics, stratified into four groups: the AKI group, the overall non-AKI group and the subgroups of 92 drafted (control of AKI patients) and 49 non-drafted non-AKI patients. AKI patients were older, had a higher frequency of heart disease and hypertension, developed sepsis more often, more frequently used mechanical ventilation and vasoactive drugs, had a higher frequency of coagulopathy and liver dysfunction, had a more positive total FB, and had higher mortality than the drafted non-AKI group, which had a lower reference SCr and a higher UO. The clinical and demographic characteristics of the 92 drafted non-AKI patients and 49 non-drafted non-AKI patients were similar.

**Figure 2:**
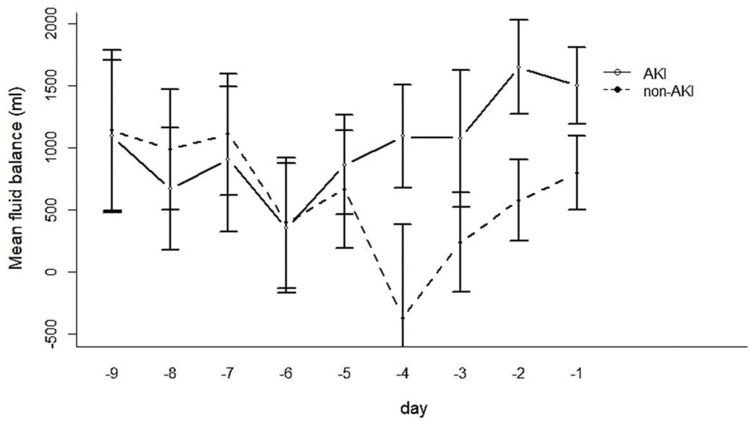
Mean fluid balance nine days before acute kidney injury (AKI) and at the corresponding time in the control group (non-AKI).

The FB of the AKI group four days before the development of AKI diverged strikingly from that of the drafted control group at the corresponding time (Figure 2).

The AKI group exhibited a significantly higher FB preceding AKI diagnosis, and there was a clear increase in the frequency of AKI in parallel with the increasing FB quartiles (Table 2).

### Logistic regression analysis

#### FB as a continuous variable and AKI

**Figure 3:**
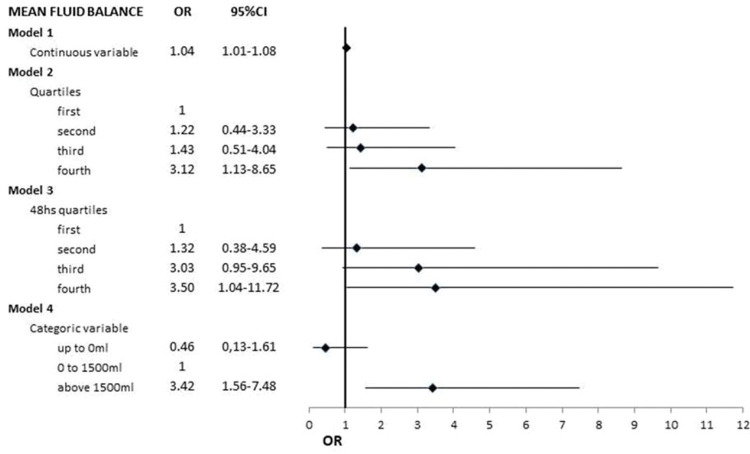
Multiple logistic regression models of the association between fluid balance (independent variable) and acute kidney injury (AKI) (dependent variable).

Comparing FB as a continuous variable up to the detection of AKI with FB during the corresponding days in the drafted controls, we found that each 100-ml increase in FB was associated with a 4% increase in the odds for subsequent AKI detection (OR 1.04; 95% CI 1.01 to 1.08) (Figure 3).

The models tested were as follows: model 1: mean fluid balance (continuous variable) until acute kidney injury and at the corresponding time in the control group (non-AKI); model 2: fluid balance on days preceding acute kidney injury and at the corresponding time in the control group (non-AKI) stratified into quartiles; model 3: the fluid balance on the two days preceding acute kidney injury and at the corresponding time in the control group (non-AKI) stratified into quartiles; model 4: mean fluid balance categorized into < zero/day, zero to 1 500 ml/day and >1 500 ml/day.

#### FB as a categorical variable and AKI

##### FB quartiles and AKI.

We aggregated the FB results preceding AKI and at the corresponding time in the control group and estimated the cut points to categorize them into quartiles: first quartile, up to -46 ml/24h; second quartile, >-46 up to 882 ml/24h; third quartile, >882 up to 1793 ml/24h; and fourth quartile, >1793 ml/24h). Patients in the fourth FB quartile had higher odds for developing AKI (OR 3.12; 95% CI 1.13 to 8.65) than patients in the first quartile. Similar results were found in a comparison of the FB quartiles in the two days before AKI (48h FB quartile) with the corresponding days in the drafted controls without AKI. Patients in the fourth 48h FB quartile had higher odds of developing subsequent AKI than patients in the first 48h FB quartile (OR 3.5; 95% CI 1.04 to 11.72) (Figure 3).

FB categorized as <zero, from zero to 1,500 and >1,500 ml/24h and AKI. A FB>1,500 ml/24h was associated with a higher OR for subsequent AKI detection than a FB of zero to 1,500 ml/24h (OR 3.4; 95% CI 1.56 to 7.48) (Figure 3).

#### Sensitivity analysis including the transient AKI patients

The sensitivity analysis with transient AKI patients showed a lower FB in the total time preceding AKI (734±1357 ml *versus* 1582±1318 ml, p=0.003) and 48h before AKI (674±1436 ml versus 1500±1278 ml, *p*=0.016) than the group of 92 AKI patients. Transient AKI patients evolved with significantly lower UO (1441±654 ml versus 1884±1093 ml, *p*=0.009), lower APACHE II score (18.0±5.7 *versus* 21.6±8.0, *p*=0.021), less coagulopathy and less need for mechanical ventilation (3.4% *versus* 19.6%, *p*=0.041 and 58.6% *versus* 94.6%, *p*<0.001, respectively) than the AKI group. Transient AKI patients developed AKI earlier in their ICU stay (2 (1-3) days *versus* 3 (2-8) days (*p*=0.005)), had a shorter ICU stay (5.9±2.3 *versus* 11.3±5.5 days, *p*<0.001), and a tendency toward lower mortality than AKI patients (41.4% *versus* 59.8%, *p*=0.082).

Logistic regression was also performed while including the 29 patients with transient AKI and 29 non-AKI patients from among the 49 non-drafted non-AKI patients in the main analysis. There was no association between FB and subsequent development of AKI after transient AKI inclusion when FB was evaluated as a continuous variable or categorized by its quartiles. In the model that included FB categorized as <zero, zero to 1,500 (reference) and >1,500 ml/24h, there was a positive association of FB>1,500 ml/24h with AKI detection (OR 2.2; 95% CI 1.14-4.19). When polynomial regression was performed with FB>1,500 ml/24h, there was a positive association of FB>1,500 ml/24h with AKI, i.e., those included in the main analysis (OR 2.4; 95% CI 1.22-4.84), but not with the transient AKI group (OR 1.7; 95% CI 0.57-5.05).

##### Mixed-effect model

**Figure 4:**
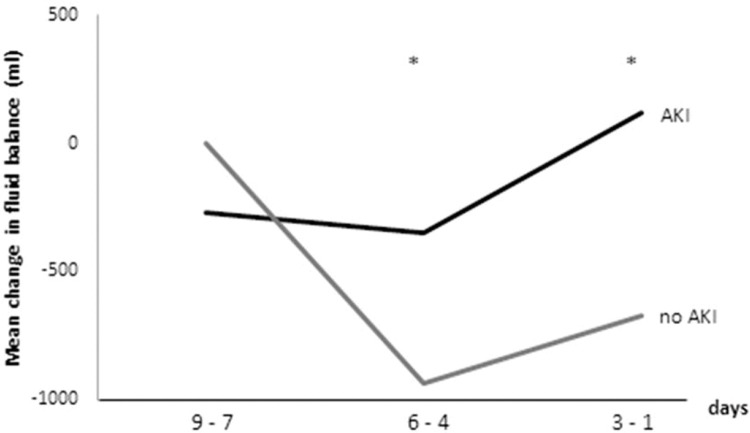
Mean change in fluid balance, according the linear mixed effects model. Note that at days -6 to -4 and -3 to -1, the fluid balance diverges significantly between the AKI and non-AKI groups.

Table 3 and Figure 4 show the results of the linear mixed effects model, comparing non-AKI and AKI patients with the same values for the adjusted variables included in the model (i.e., same age, sex, APACHE score, etc.) and taking the mean FB on days -9 to -7 in the non-AKI group as reference. The regression model showed a similar FB on days 7 to 9 before AKI and the corresponding time in the control group. The interaction between time and the presence of AKI yielded a higher FB in the AKI group than the non-AKI controls, which was significant for days 4 to 6 and even greater for days 1 to 3 before AKI.

In the sensitivity analysis, using the mean FB 4 to 6 days before AKI as reference, increased FB in the AKI group with time (1 to 3 days) was also observed.

##### Adjustment of creatinine by positive FB

When creatinine was adjusted for positive FB (26), 20 patients (8.5%) migrated from the non-AKI group to the AKI group, which had no impact on the magnitude of the association between positive FB and AKI.

### DISCUSSION

In this study, a positive FB was clearly and independently associated with AKI and preceded AKI detection by four days.

We found only four previous studies assessing the association between FB and AKI as a primary outcome (13-16). In a post hoc analysis of 100 adult cardiovascular surgery patients, a highly positive FB within the first postoperative 24h was associated with a significantly elevated AKI risk. The number of patients in the highly positive FB group was small (only 10) (13). A prospective observational study (14) stratified 90 adult cardiac surgery patients into FB quartiles from the intraoperative period until 48h postoperatively. AKI incidence was significantly higher in the fourth quartile and being in the fourth quartile was independently associated with increased odds for AKI. A secondary analysis of a prospective observational study in 98 pediatric cardiac surgery patients (15) showed that children with an early fluid overload had approximately seven times the risk for developing AKI on any post surgery day. Finally, a retrospective observational study on 121 liver transplant patients showed that positive FB in the first four postoperative days was independently associated with the development of AKI and the need for continuous renal replacement therapy (16).

Our study has significant differences from the studies described above. It was prospective and specifically designed to test the association between a positive FB and subsequent detection of AKI as a primary outcome. It assessed clinical and surgical patients in a general ICU, allowing better generalization of the results. The KDIGO AKI definition was used, considering both SCr and UO criteria, in contrast with the majority of the previous studies, which used only the AKIN or RIFLE SCr criterion (13-15) to identify AKI.

Our follow-up was not restricted to 24 to 48h, as in some of the previous studies, which is particularly important since half of the patients developed AKI after three days in the ICU. Our sample size was larger than that of the other studies, and our control group had the same exposure time to the variable FB as the AKI group, which was not done previously (18-25). Finally, we studied FB in different ways and with different thresholds.

The temporal relationship between positive FB and AKI development suggests that quiescent kidney dysfunction or subclinical AKI, not yet diagnosed by SCr or UO changes, might be present in cases with positive FB. Confirmation of these data would have important clinical applications. It might imply the inclusion of a positive FB as a diagnostic criterion for AKI along with SCr and UO, and it might reinforce that patients with a positive FB must have their SCr corrected by correcting the FB (26). Specific maneuvers might be implemented to spare this group of patients from additional kidney injury, as any patient is at a high risk for AKI.

Consistent with the concept that a positive FB may be an early marker for renal impairment, Basu et al. (27) proposed the term “renal angina” in critically ill children. Renal angina was characterized as a high-risk condition for AKI, with the presence of fluid overload, and not necessarily with increased SCr or UO reduction. The authors created the renal angina index (RAI), composed of clinical criteria, changes in SCr or estimated creatinine clearance and the percentage of fluid overload, and they validated it in ICU pediatric cohorts (28,29). A RAI ≥8 on ICU admission was associated with a higher risk for AKI development on the third ICU day (28,29). RAI was assessed in one large cohort of critically ill adult patients, and the results were consistent with the performance of renal angina in children (30).

Our study has limitations. This was a single-center study. Its observational design did not allow a causal relationship between a positive FB and AKI to be established. Though the mixed effect model was able to show a significant change in FB with time and a significant interaction with a higher FB in the AKI group, the mean FB in the time used as a reference (days -7 to -9) was estimated in fewer than half of the patients, as the median time to AKI was three days. However, when a mean FB of 4 to 6 days before AKI was used as a reference, increased FB in the AKI group over time (1 to 3 days) was also observed. Another limitation is the lack of data on the use of diuretics in both groups with and without AKI.

In conclusion, this prospective, controlled, observational study showed a significant association between positive FB and subsequent development of AKI, suggesting that positive FB might be considered an early biomarker for AKI in critically ill patients.

## AUTHOR CONTRIBUTIONS

Ávila MON was responsible for the conceptualization, data curation, formal analysis, funding acquisition, investigation, methodology, project administration, resources, validation, visualization, manuscript original drafting, editing and review. Rocha PN was responsible for the formal analysis, methodology, visualization, manuscript writing, editing and review. Perez CA and Faustino TN were responsible for the data curation, investigation and visualization. Batista PBP - was responsible for the data curation, resources and visualization. Yu L was responsible for the methodology, supervision, visualization and manuscript writing, editing and review. Zanetta DMT was responsible for the data curation, formal analysis, methodology, visualization, manuscript original drafting, editing and review. Burdmann EA was responsible for the conceptualization, formal analysis, methodology, validation, visualization, manuscript writing, editing and review.

## Figures and Tables

**Table 1:** Clinical characteristics of the overall population assessed, acute kidney injury group, overall non-acute kidney injury group, 92 drafted non-acute kidney injury group and 49 non- drafted non-acute kidney injury group.

	Total	AKI		Non-AKI	Non-AKI		
			Total	Drafted	Non-drafted		
	n = 233	n = 92	n = 141	n = 92	n = 49	*p* a	*p* b
Demographic data							
Age (years)	60±20*	64±19	57±20	58±20	55±20	0.039	0.300
Male (%)	50	51	50	50	49	1.000	0.908
Comorbidities (%)							
DM	15	19	13	12	14	0.305	0.693
Hypertension	38	46	33	30	39	0.048	0.317
Neoplasia	25	20	29	30	27	0.125	0.627
Heart Disease	20	38	7	8	8	<0.001	0.907
Organ Dysfunction (%)							
Sepsis	87	95	82	82	84	0.005	0.750
MV	78	95	67	73	57	<0.001	0.059
VAD	53	70	42	47	33	0.003	0.106
Coagulopathy	10	20	4	7	0	0.015	0.092
Liver dysfunction	12	21	6	8	2	0.019	0.262
AKI	40	100	-	-	-	-	-
AKI stage (%)							
KDIGO 1		60					
KDIGO 2		18					
KDIGO 3		22					
Renal data							
Mean FB (ml/24h)^c^	834±847	1190±858	600±756	554±687	687±871	0.027	0.146
FB until AKI (ml/24h)	-	1582±1318	-	846±1035	-	<0.001	-
Urinary output (ml/24h)	2329±1180	1884±1093	2620±1148	2664±1214	2538±1018	<0.001	0.538
SCr reference (mg/dl)	0.7±0.5**	1.0±0.6	0.6±0.2	0.6±0.2	0.6±0.2	<0.001	0.893
Death (%)	37	59	22	24	18	<0.001	0.449

* Age range from 18 - 98 years;

** SCr range from 0,1-3,9 mg/dl;

a AKI x total non-AKI;

b Drafted x Non-drafted.

**Table 2:** Fluid balance in patients with acute kidney injury and the drafted non-acute kidney injury group.

	AKI	Drafted non-AKI	
	(n=92)	(n=92)	*p*
Mean FB before AKI (ml/day)^a^	1582±1318	846±1035	<0.001
FB Quartiles (ml/day)^b^	%	%	
First (FB ≤-46)	16.3	33.7	0.001
Second (FB >-46 up to 882)	20.7	29.3	
Third (FB >883 up to 1 793)	27.2	22.8	
Fourth (FB >1 793)	35.9	14.1	
Total	100	100	
48 h FB Quartiles (ml/day)^c^	%	%	
First (FB ≤147)	13.5	35.1	0.001
Second (FB >147 up to 1016)	21.6	29.7	
Third (FB >1016 up to 1877)	28.4	21.6	
Fourth (FB >1877)	36.5	13.5	
Total	100	100	
**Categoric variable:**			
Mean FB <0 ml/day (%)	6.5	15.2	<0.001
0≤Mean FB≤1500 ml/day (%)	39.1	66.3	
FB > 1500 ml/day (%)^d^	54.3	18.5	
Total	100	100	

FB: fluid balance.

a Mean FB to AKI (ml/day) = mean FB until AKI development in the cases against the mean FB of the corresponding time in their controls (drafted non-AKI).

b FB quartile (ml/day) = distribution of patients and controls (drafted non-AKI) by FB quartiles (ml/day) estimated with FB in the days preceding AKI development in the patients and the corresponding days in the controls.

c 48h FB quartile (ml/day) = distribution of patients and controls (drafted non-AKI) by FB quartiles (ml/day) estimated with the FB in the 48 h before AKI development in the patients and the corresponding days in the controls.

d Mean FB > 1500 ml = mean FB in the days preceding AKI development in the patients and the corresponding days in the controls (drafted non-AKI).

**Table 3:** Linear mixed effect model: Fixed effects of time before acute kidney injury on fluid balance.

	Model parameter estimates^a^,^b^	SE	*p*-value^c^
6-4 days^d^	-935	260	<0.001
3-1 days^d^	-671	238	0.006
AKI	-272	320	0.398
AKI x 6-4 days^e^	855	368	0.022
AKI x 3-1 days^e^	1061	334	0.002

AKI: acute kidney injury; SE: standard error;

a ml/day;

b linear mixed effect model, adjusted for sex, age, APACHE II score, presence of sepsis, heart disease, liver dysfunction, requirement for mechanical ventilation and use of vasoactive drugs;

c Wald test;

d reference days 7 - 9 before AKI;

e interaction between the time period and the presence of AKI.
